# Supplementary material for: Serious Life Events in People with Visual Impairment Versus the General Population
Source: Int J Environ Res Public Health. 2021 Nov 2;18(21):11536. doi: 10.3390/ijerph182111536 (PMC8583190; doi:10.3390/ijerph182111536)
Supplement: Supplementary file 1 [file ijerph-18-11536-s001.zip › ijerph-1310909-supplementary.pdf]

Online supplementary material

Systematic literature search

We have performed two types of systematic literature searches to find relevant articles about traumatic events and posttraumatic stress in people with visual impairment. The first search was performed in April 2017 in the databases MEDLINE, EMBASE, PsycINFO, Web of Science, CINAHL, and Cochrane Libraries. The search included multiple terms relating to traumatic events (e.g., violence, traumatic event*, disaster*, accident*), posttraumatic stress (e.g., posttraumatic stress disorder*, posttraumatic reaction*, disaster mental health), and visual impairment (e.g., visual* impair*, vision disorder*, blindness, and vision loss). The search were limited in terms of publication status (peer-reviewed articles), publication date (1980–2017), and language (English or Scandinavian). We also screened citations and reference list of the identified articles and performed free-hand searches in Google Scholar. Of the 3492 records screened, 11 articles were identified as relevant [1].

In the second review, we searched MEDLINE, EMBASE, PsycINFO, and Web of Science in November 2019 for relevant articles. The search did not have any restrictions on publication status, date, or language. We included the following terms (and synonyms or closely related words) as index or free-text words in the searches: 'Posttraumatic stress', 'Visually Impaired Persons', and 'Low vision'. We also screened citations and reference list of the identified articles and performed free-hand searches in Google Scholar. Of the 648 records identified, 13 articles were deemed eligible and included in the review [2].

Reference list

1. Brunes, A.; Hansen, M.B.; Heir, T. Post-traumatic stress reactions among individuals with visual impairments: a systematic review. *Disabil. Rehabil.* **2018**, *39*, 691–696.

2. van der Ham, A.J.; van der Aa, H.P.; Brunes, A.; et al. The development of posttraumatic stress disorder in individuals with visual impairment: a systematic search and review. *Ophthalmic. Physiol. Opt.* **2021**, *41*, 331–341.

Results from supplementary analyses

Table S1 to S6 present results from supplementary analyses.

Table S1. Lifetime exposure to serious life events in men and women of the visual impairment population (N = 736).

			**Men (N = 333)**			**Women (N = 403)**	
**Event categories**	**n**	**%**	**95% CI**	**n**	**%**	**95% CI**	**M/W ratio¤**
Natural disaster	31	9.3	6.2-12.4	40	9.9	7.0-12.9	0.94
Fire or explosion	40	12.0	8.5-15.5	42	10.4	7.4-13.4	1.15
Transport accident	72	21.6	17.2-26.1	94	23.3	19.2-27.5	0.93
Other serious accident	55	16.5	12.5-20.5	75	18.6	14.8-22.4	0.89
Exposure to toxic substance	31	9.3	6.2-12.4	10	2.5	1.0-4.0	3.72***
Physical assault	55	16.5	12.5-20.5	71	17.6	13.9-21.3	0.94
Assault with weapon	25	7.5	4.7-10.3	28	7.0	4.5-9.4	1.07
Sexual assault	8	2.4	0.8-4.1	71	17.6	13.9-21.3	0.14***
Other unwanted sexual experience	11	3.3	1.4-5.2	76	18.9	15.0-22.7	0.18***
Combat	18	5.4	3.0-7.8	21	5.2	3.0-7.4	1.04
Captivity	4	1.2	0.0-2.4	9	2.2	0.1-3.7	0.55
Life-threatening illness or injury	97	29.1	24.2-34.0	94	20.8	16.9-24.8	1.40**
Illness or injury causing VI	133	39.9	34.7-45.2	130	32.2	27.7-36.8	1.24
Severe human suffering	39	11.7	8.3-15.2	44	10.9	7.9-14.0	1.07
Witness violent death	9	2.7	1.0-4.4	16	4.0	2.1-5.9	0.68
Sudden accidental death	13	3.9	1.8-6.0	9	2.2	0.1-3.7	1.77
Causing serious injury or death to others	6	1.8	0.0-3.2	2	0.5	0.0-1.2	3.60
Other very stressful events	49	14.7	10.9-18.5	95	23.6	19.4-27.7	0.62**
At least one event	248	74.5	69.8-79.2	307	76.2	72.0-80.3	0.98

Notes. *p<0.05, **p<0.01, ***p<0.001

Table S2. Associated factors of fire or explosion in the visual impairment population (N = 736) estimated using regression analysis.

Covariates	Unadjusted PR (95% CI)	Fully adjusted PR (95% CI)
Age (continuous)	**1.13 (1.01–1.27)**	1.11 (0.96–1.28)
Male gender (ref. females)	1.15 (0.77–1.73)	1.11 (0.74–1.66)
Lower education (ref. higher education)	1.01 (0.67–1.53)	1.01 (0.67–1.56)
Unemployed (ref. employed/under education)	1.29 (0.84–1.99)	0.95 (0.57–1.59)
Unmarried (ref. married/cohabitant)	1.03 (0.69–1.56)	1.11 (0.71–1.73)
Self-reported blindness (ref. moderate-to-severe)	1.37 (0.89–2.12)	1.43 (0.91–2.23)
Nature of vision loss (ref. congenital)		
Childhood	**2.05 (1.19–3.50)**	**2.04 (1.19–3.50)**
Adulthood	**1.75 (1.08–2.85)**	1.67 (0.96–2.93)
Having other impairments (ref. no impairments)	1.31 (0.87–1.98)	1.19 (0.77–1.89)

Notes. PR: prevalence ratio; Ref: reference; Text in bold indicates statistical significance.

Table S3. Associated factors of serious accidents in the visual impairment population (N = 736) estimated using regression analysis.

Covariates	Unadjusted PR (95% CI)	Fully adjusted PR (95% CI)
Age (continuous)	0.90 (0.82–0.99)	0.93 (0.83–1.04)
Male gender (ref. females)	0.89 (0.65–1.22)	0.86 (0.63–1.18)
Lower education (ref. higher education)	0.81 (0.59–1.11)	0.74 (0.54–1.04)
Unemployed (ref. employed/under education)	0.76 (0.55–1.03)	0.71 (0.49–1.02)
Unmarried (ref. married/cohabitant)	1.22 (0.89–1.67)	1.19 (0.86–1.64)
Self-reported blindness (ref. moderate-to-severe)	0.93 (0.64–1.34)	0.93 (0.64–1.35)
Nature of vision loss (ref. congenital)		
Childhood	1.10 (0.74–1.65)	1.04 (0.69–1.52)
Adulthood	0.91 (0.64–1.30)	1.03 (0.71–1.54)
Having other impairments (ref. no impairments)	**1.85 (1.36–2.52)**	**2.13 (1.54–2.94)**

Notes. PR: prevalence ratio; Ref: reference; Text in bold indicates statistical significance.

Table S4. Associated factors of sexual assaults in the visual impairment population (N = 736) estimated using regression analysis.

**Covariates**	**Unadjusted PR (95% CI)**	**Fully adjusted PR (95% CI)**
Age (continuous)	0.90 (0.81–1.01)	0.83 (0.73–0.94)
Male gender (ref. females)	–	–
Lower education (ref. higher education)	1.00 (0.66–1.52)	0.73 (0.48–1.12)
Unemployed (ref. employed/under education)	**1.73 (1.08–2.77)**	**2.08 (1.21–3.55)**
Unmarried (ref. married/cohabitant)	**2.46 (1.53–3.97)**	**2.35 (1.40–3.94)**
Self-reported blindness (ref. moderate-to-severe)	1.37 (0.88–2.14)	1.40 (0.89–2.18)
Nature of vision loss (ref. congenital)		
Childhood	1.34 (0.79–2.27)	1.25 (0.72–2.17)
Adulthood	1.02 (0.63–1.66)	1.30 (0.75–2.26)
Having other impairments (ref. no impairments)	**1.81 (1.19–2.73)**	1.47 (0.95–2.28)

Notes. PR: prevalence ratio; Ref: reference; –: not applicable; Text in bold indicates statistical significance.

Table S5. Associated factors of life-threatening illness or injury in the visual impairment population (N = 736) estimated using regression analysis.

**Covariates**	**Unadjusted PR (95% CI)**	**Fully adjusted PR (95% CI)**
Age (continuous)	**1.15 (1.07–1.24)**	1.07 (0.98–1.16)
Male gender (ref. females)	**1.40 (1.08–1.80)**	**1.37 (1.07–1.74)**
Lower education (ref. higher education)	0.85 (0.66–1.09)	0.79 (0.61–1.01)
Unemployed (ref. employed/under education)	**1.66 (1.25–2.21)**	1.21 (0.87–1.68)
Unmarried (ref. married/cohabitant)	1.03 (0.80–1.33)	1.11 (0.86–1.42)
Self-reported blindness (ref. moderate-to-severe)	1.26 (0.96–1.65)	**1.32 (1.01–1.73)**
Nature of vision loss (ref. congenital)		
Childhood	**2.01 (1.42–2.85)**	**1.78 (1.26–2.53)**
Adulthood	**2.02 (1.49–2.74)**	**1.73 (1.24–2.42)**
Having other impairments (ref. no impairments)	**2.09 (1.63–2.69)**	**1.87 (1.44–2.42)**

Notes. PR: prevalence ratio; Ref: reference; Text in bold indicates statistical significance.

Table S6. Associated factors of severe human suffering in the visual impairment population (N = 736) estimated using regression analysis.

**Covariates**	**Unadjusted PR (95% CI)**	**Fully adjusted PR (95% CI)**
Age (continuous)	0.96 (0.87–1.07)	**0.87 (0.76–1.00)**
Male gender (ref. females)	1.07 (0.72–1.61)	1.04 (0.70–1.55)
Lower education (ref. higher education)	1.03 (0.69–1.56)	1.00 (0.66–1.51)
Unemployed (ref. employed/under education)	1.12 (0.74–1.71)	0.96 (0.60–1.55)
Unmarried (ref. married/cohabitant)	1.06 (0.70–1.59)	1.07 (0.72–1.61)
Self-reported blindness (ref. moderate-to-severe)	1.07 (0.67–1.69)	1.29 (0.82–2.04)
Nature of vision loss (ref. congenital)		
Childhood	1.74 (0.98–3.10)	1.60 (0.90–2.85)
Adulthood	**2.13 (1.32–3.44)**	**2.61 (1.49–4.55)**
Having other impairments (ref. no impairments)	**1.81 (1.21–2.71)**	**1.71 (1.12–2.62)**

Notes. PR: prevalence ratio; Ref: reference; Text in bold indicates statistical significance.
